# Surface Electromyography Study Using a Low-Cost System: Are There Neck Muscles Differences When the Passenger Is Warned during an Emergency Braking Inside an Autonomous Vehicle?

**DOI:** 10.3390/s21165378

**Published:** 2021-08-09

**Authors:** Silvia Santos-Cuadros, Sergio Fuentes del Toro, Ester Olmeda, José Luis San Román

**Affiliations:** 1 Mechanical Engineering Department, Universidad Carlos III de Madrid, Avda. de la Universidad 30, 28911 Leganés, Spain; sfuentes@ing.uc3m.es (S.F.d.T.); eolmeda@ing.uc3m.es (E.O.); jlsanro@ing.uc3m.es (J.L.S.R.); 2 Institute for Automotive Vehicle Safety (ISVA), Universidad Carlos III de Madrid, Avda. de la Universidad 30, 28911 Leganés, Spain

**Keywords:** surface electromyography, low-cost, autonomous bus, cervical muscles response, injury, emergency braking

## Abstract

Deaths and serious injuries caused by traffic accidents is a concerning public health problem. However, the problem can be mitigated by the Autonomous Emergency Braking (AEB) system, which can avoid the impact. The market penetration of AEB is exponentially growing, and non-impact situations are expected to become more frequent. Thus, new injury patterns must be analysed, and the neck is particularly sensitive to sudden acceleration changes. Abrupt braking would be enough to be a potential risk for cervical spine injury. There is controversy about whether or not there are differences in cervical behaviour depending on whether passengers are relaxed or contract their muscles before the imminent accident. In the present manuscript, 18 volunteers were subjected to two different levels of awareness during an emergency braking test. Cervical muscles (sternocleidomastoid and trapezius) were analysed by the sEMG signal captured by means of a low-cost system. The differences observed in the muscle response according to gender and age were notable when passengers are warned. Gender differences were more significant in the post-braking phase. When passengers were relaxed, subjects older than 35 registered higher sEMG values. Meanwhile, when passengers contract their muscles, subjects who were younger than or equal to 35 years old experienced an increment in the values of the sEMG signals.

## 1. Introduction

Deaths and serious injuries caused by traffic accidents represent a serious public health problem with broad social and economic consequences. However, contrary to what many people believe, accidents are preventable. Protection provided by vehicles through an Assistance and Driving Aid System (ADAS) can help reduce this problem. Among these systems, the Autonomous Emergency Braking (AEB) system stands out. This system identifies imminent collisions based on the recognition of objects placed in front of the vehicle by means of a camera and reacts automatically by activating the brakes. In this way, AEB can avoid certain types of collisions. Considering the obligations established by the regulations to install AEB in all new vehicles, these emergency braking and, in some cases, non-impact situations are expected to become more and more frequent. This implies new patterns of injury. The fact that an impact does not happen does not involve no injuries in the occupants [1,2,3,4,5]. Sudden braking would be enough to be a potential risk for cervical spine damage, since neck is particularly sensitive to sudden changes in acceleration. The role of muscles is fundamental in cervical injury. Indeed, there are several studies [6,7,8] that affirm that muscles are the main location of neck injury, especially in low-speed cases.

During dynamic loads, muscles fibres of the neck contract to stiffen the head–neck complex in order to reduce spinal column motion. The period during which the muscle is contracted varies depending on the passenger awareness [9]. Those people who are warned previously about the event and know when this event will occur contract their muscles prior to impact. By contrast, unaware occupants contract cervical muscles as a reflex act during impact. There are researchers who affirm that in the event of a possible accident, there are no awareness-related differences [7], and then being relaxed could be better; while others, on the contrary, suggest that it is better to contract the muscles before the accident [10], because the relative movement between the different parts of the body is smaller. The question then arises as to which situation implies less risk of cervical injury in an emergency braking case. Be relaxed? Or contract the muscles before the braking? Therefore, considering the design characteristics of a vehicle safety system such as AEB, which situation implies a lower risk related to cervical injury? Should the vehicle warn that an imminent braking is going to happen, making it possible to contract muscles beforehand? Or, on the contrary, would it be better if the system does not warn about it and the muscles of the passenger were relaxed before sudden braking?

Finding whether there are differences in cervical muscle behaviour depending on whether the muscles are relaxed or contract before emergency braking is our main objective. For this, two different emergency braking tests will be carried out. In the first place, a test will be performed in which passengers will not be notified of the exact moment in which the emergency braking will take place, thus ensuring that they keep their muscles relaxed. Next, a second test will be carried out where passengers will be alerted to the moment at which the emergency braking is going to take place, so that they contract the muscles previously. On the other hand, there are research areas focused on the customised design of road safety based on gender and age [11,12]. Women have a 47% higher risk of serious injury in a car crash than men and five times higher risk of whiplash injury [13]. However, most biomechanical models used in laboratories do not consider the anthropometric differences between women and men. Therefore, simulated crashes do not predict well female injuries. The overall effectiveness of occupant safety devices is biased toward male occupants. To the above, it should be added that older people are considered vulnerable users in road safety, since their body offers less resistance to impacts, so the probability of suffering a serious injury is higher as age increases. For this reason, analysing possible gender and age differences in cervical muscle behaviour is the other main aim of our work.

In order to analyse muscle behaviour, it is essential to have the maximum muscle biofidelity as possible. There are some biomechanical models such as cadavers, dummies, or computational models that do not have sufficient fidelity in the muscular response. This absence of biofidelity of these models can be solved by collecting data from human subjects. This factor is precisely one of the strengths of this study, where 18 volunteers participated.

In this work, the technique used to assess the cervical muscle response is surface electromyography (sEMG). During an emergency braking, the neck injury mechanism implies a sudden forward motion of the head, followed by another abrupt rearward movement. Considering this and similar studies in the literature [14,15,16,17], the trapezius (TRP) and sternocleidomastoid (SCM) were selected as target neck muscles. The SCM supports most of the dynamic load of the cervical area during rear impacts, while the TRP muscle bears most of the load in frontal impacts. There are other cervical muscles, such as the Scalenus muscle or Rectus Capitis Lateralis muscle, but they are deep muscles. If we wanted to properly assess the response of these deep muscles, the best option would be through intramuscular or needle electrodes. This would involve the need of researchers specialised in medicine, to which it should be added that this technique causes a greater probability of infection in the subject. It must be emphasised that neck injuries caused by non-collision accidents remain a meaningful health problem with a significant social cost [18,19]. Although these types of traffic accidents do not culminate in a final impact with another vehicle or obstacle on the road, they can involve significant levels of deceleration, translated into high loads that must be resisted by different anatomical regions. Among them, the cervical area stands out for being a very sensitive area to sudden changes in acceleration.

Furthermore, these musculoskeletal disorders (such as the controversial whiplash injury) are expected to continue growing due to the increase of the AEB systems in vehicles as well as the autonomous vehicles where these systems are also installed. Therefore, there is a need for performing new injury pattern studies where the traffic scenario can be different since the emergency braking system would avoid the impact. 

Bearing in mind all the above, the hypotheses considered for the present work are the following:

- First hypothesis: the passenger awareness of an emergency braking involves differences in the cervical muscle response.
- Second hypothesis: there are differences in cervical muscle response during an emergency braking due to gender and age.

Answering these assumptions is the main aim of this study.

## 2. Materials and Methods

### 2.1. Experiment Scenarios

18 Volunteers were subjected to two situations of automatic emergency braking in this work, simulating a sub-injury level situation to avoid damage to the subjects. In these braking tests, volunteers were travelling inside a real autonomous vehicle with an AEB system installed. Subjects were positioned seated in the direction of travel. In one of the braking tests, the volunteers did not know when the sudden braking would happen, which allowed the neck muscles to be relaxed. On the other braking test, the volunteer was warned before the braking, so that the subject could contract the muscles before the braking. In this way, the conclusions drawn by this study would contribute to understand new possible road traffic scenarios when autonomous mobility becomes a reality. Moreover, findings could help to design more adequate and safer road safety system in vehicles for all types of passengers. 

A call to participate in the experiment was sent to a group of people, and 18 of them accepted to be part of this study. Fifty-six percent of the volunteers were males and 44% were females.

Participation in this research was completely voluntary. Prior to its acceptance, the aim of the study, the methodology to be followed, and the instrumentation to be used, as well as the tests to be carried out, were explained to the interested participants. After receiving their interest in participating, they signed an informed consent. However, any time, after or before signing this document, the volunteer could decide to change his/her mind and not participate. In other words, all of them can leave the study in any step. The protocol followed in this work meet the requirements for research involving human subjects according to the Declaration of Helsinki.

All tests were performed within the admitted physiological limits, so the volunteer was not subjected to levels of deceleration that could cause injuries. Nevertheless, if, during the trial, the participant experienced any kind of discomfort or pain, they were required to immediately notify the research team, and their participation was interrupted instantly. Therefore, regarding the inclusion and exclusion criteria, to be able to participate in this study, volunteers must be in good health and not have any type of injury that could be aggravated by participating in these trials, such as neck injuries.

The volunteers were between 22 and 54 years old (31.9 ± 8.8 years). Their weight was above 47 and below 90 kg (66.3 ± 13.1 kg), and their height between 154 and 189 cm (170.8 ± 9.9 cm).

**Figure 1:**
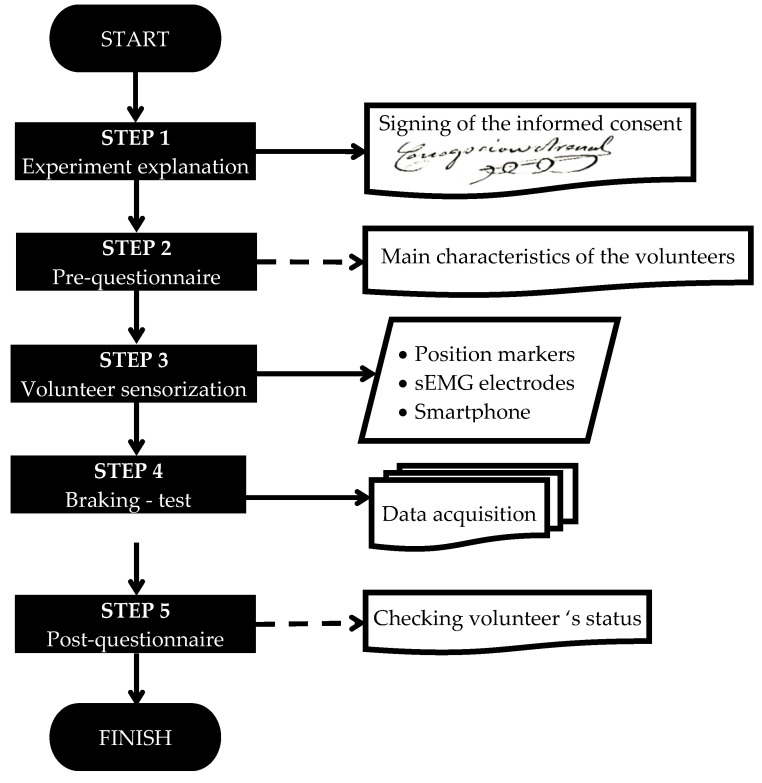
Steps to complete the emergency braking test. Based on [20].

As it was previously said, the experiment was split into different steps that are represented in the following figure (Figure 1).

**Figure 2:**
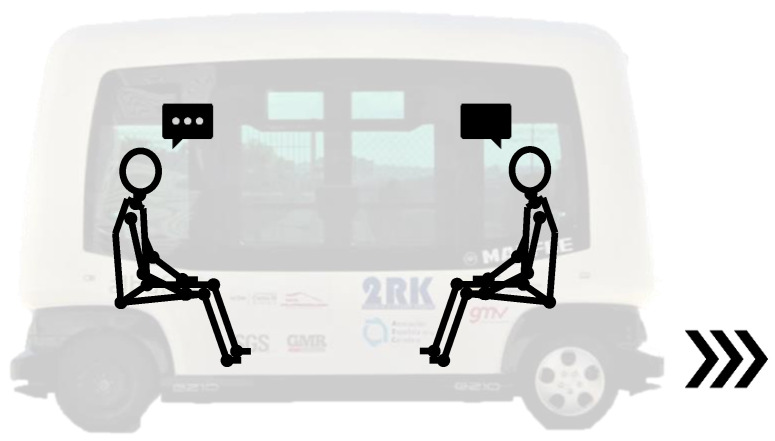
Emergency Braking Test 1 (BT1) schema [20].

- Experiment explanation (step 1): The first step was designed to explain to every volunteer the experiment and to inform them about the possible risk. Once it was explained and the volunteer decided to participate, he/she signed a consent form. It is important to highlight that finishing the experiment was not compulsory, and each volunteer could stop the experiment at any moment and leave it.
- Pre-questionnaire (step 2): The first questionnaire was delivered before the emergency braking test and was planned to gather the anthropometric information of the volunteers, such as the height, weight, gender, or age, among others. In addition, this test was designed with the idea of detecting if the volunteer was unhealthy and could have some injury during the braking emergency test.
- Volunteer sensorisation (step 3): By means of a palpation test [21,22], the sEMG sensors were applied to the volunteer on the trapezius (TRP) and the sternocleidomastoid (SCM) muscle. Before that, the area was shaved with a disposable razor blade and cleaned with alcohol and a sterile muslin.
- Some reflectors were additionally placed on different parts of the volunteer (head, spine, torso, shoulder, pelvis, and lower limbs) with the aim of collecting the motion of the volunteer during braking employing a high-speed camera. Braking test (step 4): The braking test consists of accelerating the autonomous bus to a constant velocity, and after that, braking the autonomous bus with the emergency braking system. It did not start until the volunteer had understood the experiment, had no more questions, and he/she was sensorised. This braking test was designed taking into account several references [2,7,15,16,23,24,25,26,27,28,29,30,31,32,33,34,35,36,37,38,39]. In total, there were two braking test scenarios: Braking Test 1 (BT1) and Braking Test 2 (BT2). Both are explained in the following paragraphs. To prevent the habituation of the muscles, each volunteer participated only one time in each braking test. Habituation can appear if it is repeated with a diminution of the 30–50% in the neck muscle EMG activity [40]
  ○ Emergency Braking Test 1 (BT1): The autonomous bus drives forward and the subject is sitting in the direction of travel (forward). Another person is sitting in front of him (Figure 2) to talk while the experiment is developed. Suddenly, the bus brakes without any kind of warning alert and the BT1 finishes.
  ○ Emergency Braking Test 2 (BT2): The autonomous bus drives forward and the subject is sitting in the direction of travel (forward). Another person is sitting in front of him (Figure 2) to talk while the experiment is developed. Suddenly, the bus brakes and there is an acoustic warning to advised about it.

In both cases, the position of the volunteer and the person who is sitting in front of him have the same position. That is to create a stress-free situation. In addition, thanks to that, the position of the head is appropriate for the experiment.

**Figure 3:**
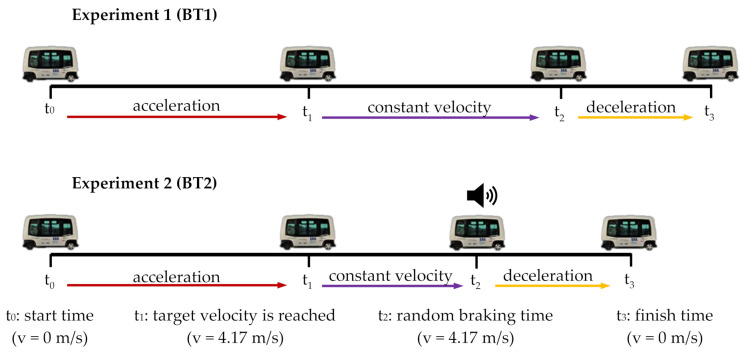
Timing of BT1 and BT2 experiments. Based on [20].

The timing of the experiment can be seen in Figure 3, where first there is an acceleration time (from t_0_ to t_1_) to reach the velocity of 4.17 m/s (15 km/h). After that, the velocity is maintained during a period between t_1_ and t_2_, when the emergency braking system is activated and the vehicle stops (t_3_).

Figure 3 shows experiments BT1 and BT2, where the unique difference is the time between t1 and t2, because the time when the autonomous bus starts braking in the BT2 experiment (t2) is randomly selected.

- Step 5 (Post-questionnaire): The last step is a questionnaire that was delivered to each volunteer to be completed after the BT1 and BT2 experiments to assure each one had not suffered any kind of injury or pain related to the experiments. In addition, this test was split into two different questioners, one delivered immediately after the test and the other one delivered one day after the experiment. After analysing the questionnaires, the authors can guarantee that none of the volunteers suffered any kind of pain or injury in the experiment.

### 2.2. Equipment

**Figure 4:**
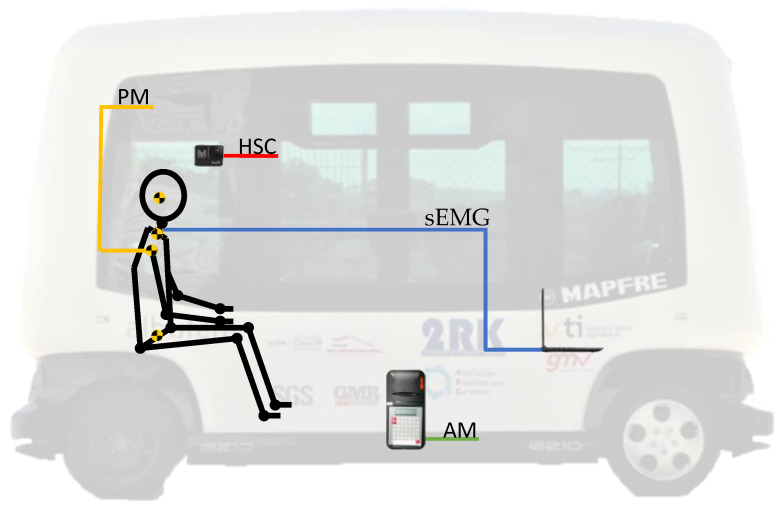
Devices used in the emergency braking test. PM: Position Markers, HSC: High-Speed Camera, AM: Accelerometer, sEMG: Sensors of Surface Electromyography. Based on [20].

The equipment used to carry out the data acquisition in the different scenarios includes the following devices (Figure 4). Figure 4 also shows the position where each one was installed inside the autonomous bus.

- Autonomous bus (EasySmile EZ10): A 6-seater autonomous bus (without driver) designed for smart mobility in private or public transport.
- Position Markers (PM): small reflective material-coated balls that provide an indicator of the passenger’s movement during the emergency braking tests.
- sEMG (surface EMG device): the device is built through an Arduino Mega board and the sEMG low-cost sensor. Both components are connected by means of various wires, and the EMG signal is captured thanks to the adhesive electrodes that must be stacked on the skin of the volunteers. The low-cost custom system needs three electrodes, one close to the middle of the muscle body, the second one lined up with the direction of the muscle fibres and close to the end of it, and the last one placed near to a bony area as a reference. The system was programmed using a personal computer by means of Simulink and Matlab [41]. It is worth mentioning that this system has previously been used in other studies with very positive results [42,43]. The technical information of the components can be found in Table 1.

- High-Speed Camera (HSC): the high-speed camera was installed parallel to the volunteer on one side of the bus. The video recorder allows us to check the evolution of the experiment and if it was properly developed. The main characteristics of the camera can be found in the following Table 2.

- Accelerometer (AM): knowing the acceleration of the vehicle in the experiment contributes to the analysis of the braking effects. To that aim, an accelerometer, with the characteristics shown in Table 3, was installed in the basement of the vehicle, close to the gravity centre.

### 2.3. Analysis Method

The analysis of the different signals gathered in the experiments was split into different steps. Firstly, all the signals were synchronised and after the sEMG timed, filtrated, and normalised. More information about the synchronisation can be found in [20], where all the steps in the process were carefully explained.

The following sections explain the working process for the EMG signal and the position markers.

#### 2.3.1. sEMG Signal

The EMG signal is normally affected by the noise that is necessary to filter before analysing the results. For that reason, the signals captured by the low-cost system from the TRP and the SCM muscles were filtered following the recommendations of other authors [44,45,46].

After the filtration, the sEMG signals were normalised [47]. Although the literature presents different ways to normalise the signals, by the MVC (Maximal Voluntary Contraction) [48] or by the maximum value reached in the experiment [47], the most appropriate for the present study was the normalisation based on the mean activation levels obtained during the task [49]. It was the most secure method for the volunteers because the first method was not only recommended for static studies [50], but also because an MVC done in a bad way could cause stress in the muscles [51] that could affect the volunteer (injury) and the results of the experiment. The second method does not include the whole sample and makes it difficult to compare between volunteers [47]. For all those reasons, the normalisation method chosen was the third one. This method combines a bad injury risk and a way to compare results [52,53].

Once the normalisation method was decided, we developed a script in Matlab [41] that includes the following signal treatment stages:

1. Filtration of the signals: Filtration was separated into two steps. Initially, we identified the background noise of the signal and later the main noise using a Fast Fourier Transform evaluation [42,54]. Once identified, two filters were considered, a bandpass Butterworth (40–100 Hz, order 4) filter to remove the main noise and the stopband Butterworth (45–55 Hz, order 4) to filter some noise placed on the 50 Hz band.
2. Results computation: the minute the signal was clean, some parameters were computed by Matlab and script written for that proposal. The script was focused on the following:
  ○ Amplitude for every muscle (TRP and SCM) and every experiment, individually.
  ○ Locating the times when the muscle works between the braking starts and finishes.
  ○ Maximum values in the sEMG signals during the emergency braking test.

#### 2.3.2. Position Markers

The position of the markers and the video recorded during the experiment inside the autonomous bus were analysed by video photogrammetry software, which resumes the position of the markers for each step. In addition, we wrote an additional script in Matlab that makes it possible to get the trajectory and relative movement of the head and torso.

Notwithstanding, this analysis is not the scope of the present work.

## 3. Results

Turning now to the experimental results, the next section explains the main results. Moreover, the amplitude and maximum values of the sEMG signals, once they had been normalised, were analysed for each volunteer in both autonomous emergency braking tests (without and with warning passengers). These values are represented by means of their average value (µ).

The results have been divided into two different phases within the emergency braking test. First, the braking phase is presented. This stage corresponds to the braking itself, that is, the period from when the vehicle begins to brake until it finally stops. Then, the post-braking phase takes place. This other stage corresponds to the period after braking, that is, from when the vehicle has stopped until the passenger stops moving. It must be considered that the subject will continue to move after the vehicle stops, due to their different inertia and the movement caused by the sudden deceleration. This would cause the passenger to continue moving until something stops the excursion, such as an obstacle or the resistance offered by muscles.

### sEMG Signals

**Figure 5:**
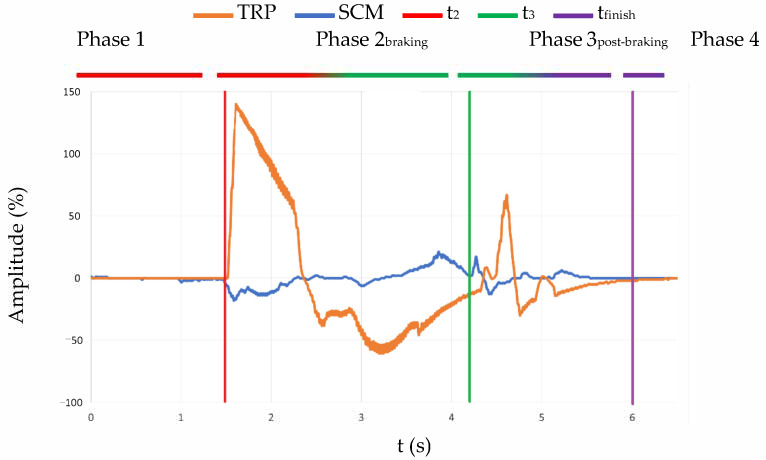
sEMG signal from the TRP and SCM muscles through a complete braking experiment.

The sEMG signals acquired were from two different muscles, an agonist and an antagonist regarding the movement. In this section, all the graphics related to the EMG signal are going to show the signal from both muscles at the same time. Therefore, it is possible to point out the contraction in every step of the experiment. An example can be seen in Figure 5. This figure shows the contraction of the muscles in the whole experiment, in which we observed different phases defined by time. The first one is what is happening before t_2_ (red line) when the volunteer is relaxed. After that comes the second phase, which is enveloped between t_2_ (red line) and t_3_ (green line) and is when we observed the contraction due to the emergency braking. Once the bus is completely stopped, that signals the third phase (between t_3_ (green line) and t_finish_ (purple line)), which the authors have decided to name post-braking, and where the bounces caused by emergency braking can be seen.

**Figure 6:**
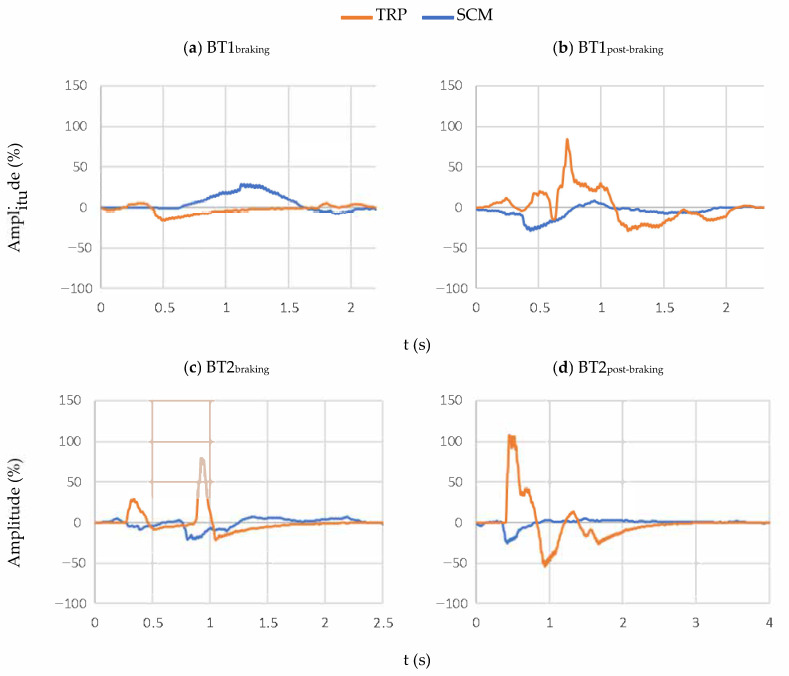
sEMG signal from the SCM and TRP muscles from one volunteer. The signal has been split according to the experiment and to the time of the braking. (**a**) BT1 braking time; (**b**) BT1 post-braking time; (**c**) BT2 braking time; (**d**) BT2 post-braking time.

For a better understanding of the results, the signals that are shown in the following lines (Figure 6) have been triggered. This provides a better definition of the different distinctive points of each experiment.

The example that is shown in Figure 6 represents, in the left column, the muscle contractions during the emergency braking (from t_2_ to t_3_), and in the right column, the muscle contractions once the bus has completely stopped (from t_3_). The signal of each muscle is represented in different colours, blue for the SCM and orange for the TRP response.

Moreover, it was evaluated whether there were differences in the cervical behaviour of the passengers according to their gender (♀ women and ♂ men) and age (younger than or equal to 35, and older than 35). This age threshold to disaggregate our sample data in two age groups was decided considering the physical and physiological changes associated with the age of 35 (the beginning of the so-called “early–middle-aged”). From this age on, people start to lose muscle mass and flexibility is reduced. We must pay special attention to the sEMG signal of the trapezius, as this muscle is essential in trying to retain the movement of the head concerning the torso when passengers travel in the direction of travel.

It was also included the µ and the P.P.O.M parameter. µ is the mean value and P.P.O.M is a variable defined by the percentage of people (♀ or ♂) whose amplitude or maximum value was higher than the mean value. In addition, P.P.O.M was calculated considering the value according to the genre of the volunteer.

It should also be noted that two different phases are analysed within the emergency braking test. On the one hand, results of the first phase of the test (Phase 2_braking_) are presented. This stage corresponds to the braking itself, that is, the period from when the vehicle begins to brake until it finally stops. On the other hand, the results of the second phase of the test (Phase 3_post-braking_) are shown. This stage corresponds to the period after braking, that is, from when the vehicle has stopped until the passenger stops moving. It must be taken into account that the subject will continue to move after the vehicle stops, due to its different inertia and the movement caused by the sudden deceleration. It will cause the passenger to continue moving until there is something that stops said excursions, such as an obstacle or the resistance offered by muscles to try not to get thrown out of the seat. 

After analysing the results, we observed that there are differences in cervical behaviour based on gender. In the case of the BT1 (without warning) (see Table 4), men experienced higher sEMG signal intensity of trapezius in both phases (braking and post-braking); while women showed a greater cervical response of SCM particularly in the post-braking stage. In any case, greater values of the sEMG signal are registered in the phase after braking for both women and men. 

In the BT2 (with a warning) (see Table 5), women suffered greater sEMG values of both cervical muscles in the braking phase, while men experienced a higher cervical response of TRP and SCM in the post-braking stage. These differences due to gender are more significant in the post-braking stage. On the other hand, when passengers are warned of the braking, it was also observed that men experienced greater values of the sEMG signal in the post-braking phase. In fact, these differences concerning the braking stage in men were more significant in BT2 than in BT1. On the contrary, women showed lower sEMG values in the post-braking phase.

Next, results are shown taking into account the age of the volunteers. The sample was divided into two age groups: less than or equal to 35 years and older than 35 years. 

When passengers have not been warned in advance of the emergency braking (BT1 test) (see Table 6), increases in the intensity of the sEMG signal of TRP muscle are observed in subjects older than 35 years of age, regardless of gender, in the post-braking phase. However, these increments are more significant in women, reaching 226% higher values for this age group. 

In the case of the braking stage, women over 35 years of age continue to experience higher sEMG signal values. However, men do not seem to experience large changes in the electromyographic signal depending on their age in the emergency braking phase. 

By including age in the analysis, it continues to be maintained that greater sEMG values are registered after stopping the vehicle (post-braking stage), independently of gender and age group. On the other hand, women suffered more activity in SCM muscle than men in both tests and for all age groups. For their part, men under or equal to 35 years experienced more TRP sEMG signal in both phases. Nevertheless, when men are older than 35, they registered lower sEMG values of TRP muscle than women. 

If we analyse the results when passengers are previously warned about emergency braking (BT2) (see Table 7), we can observe differences. Subjects younger than or equal to 35 experienced significant increases in the intensity of the sEMG signals of both cervical muscles, regardless of gender, in braking and post-braking phases. In this case (BT2), it is remarkable that women experience lower values of the sEMG signal in the post-braking phase with respect to the braking stage. While, on the contrary, men show higher values in the post-braking phase, also showing more notable differences between both stages.

In the case of the braking phase, women older than 35 continue to experience higher sEMG signal values. However, men do not seem to experience large changes in the electromyographic signal depending on their age in the emergency braking phase. 

## 4. Discussion

Deaths and serious injuries caused by traffic accidents continue to be a concerning public health problem. This can be significantly mitigated by certain Assistance and Driving Aid Systems such as the Autonomous Emergency Braking (AEB) system, which can avoid the impact. This non-impact situation implies new injury patterns. In particular, the neck is very sensitive to sudden changes in acceleration, such as those caused by emergency braking. This is the reason why, in this study, the response of two superficial cervical muscles (trapezius (TRP) and sternocleidomastoid (SCM)) from 18 volunteers was assessed by sEMG. 

Two different emergency braking tests were designed for this study, where their main difference is the awareness of the passenger about the time of emergency braking. This was decided considering controversies of previous studies where some authors did not find notable differences between warned or relaxed occupants, while other researchers suggested that it is better to contract the muscle before the accident. It should be noted that all tests were carried out at low speed to avoid any type of damage to the volunteer. Results were split according to gender and age because previous studies found important anthropometric differences that should be considered in the possible different injury patterns in traffic accidents. In fact, after analysing the results, we appreciated differences in cervical muscle behaviour based on gender and age, and depending on the braking phase.

If we focus on the results observed when passengers have not been warned of the emergency braking (BT1 test), men experienced higher sEMG signal intensity of trapezius in both phases (braking and post-braking), while women showed a greater cervical response of SCM particularly in the post-braking stage. In any case, this last phase seems to imply a greater risk of cervical injury, since higher values of the sEMG signal are reached in both men and women. This may be due to the fact that after the vehicle stops, the passenger continues to move, experiencing a series of rebounds, moving the head several times back and forth until it finally stabilises. This situation causes an overload on the neck, alternating between the performance of both cervical muscles TRP and SCM, until they manage to stop the movement of the head with respect to the torso. If we now approach the issue from the perspective of those passengers who were warned about braking (BT2 test), women suffered greater sEMG values of both cervical muscles in the braking phase, while men experienced higher cervical response of TRP and SCM in the post-braking stage. Gender differences are more significant in this last phase. The post-braking phase continues to be more harmful, since higher sEMG values are also obtained in this stage with respect to the braking phase, but now this only occurs in the case of men. On the contrary, women showed lower sEMG values in the post-braking phase in BT2. In addition, these differences with respect to the braking stage in men were more significant in BT2 than in BT1. Therefore, the post-braking phase affects the cervical behaviour more when the passenger is warned about the sudden braking. This seems to indicate that during the post-braking phase (once the first forward movement of the head has already taken place as a result of the braking itself), despite having been previously warned (and, therefore, tensing our muscles), it is much more difficult to maintain conscious control of the tension of our muscles to stabilise our movement. Men are more sensitive to these differences in the post-braking phase, while women are more sensitive in the initial braking phase.

Men show more cervical muscle activity during stabilisation (post-braking). It should be noted that there could be two explanations for these results (higher values of sEMG signals). Either their muscles suffer more (more possibility of damage), or they activate by tightening the muscles more, which translates into less relative movement of the head regarding the torso. The latter can translate into a lower risk of bone injury to the cervical vertebrae, since there is less relative movement of the head with respect to the torso. However, the fact of registering a greater electromyographic activity of the cervical muscles could also imply higher risk of muscular injury at the cervical spine. Therefore, the authors consider that more studies should be carried out to affirm which of the two situations would be more harmful globally (considering not only the risk of muscle damage but also the risk of damage to bone and other biological tissues). To do this, the authors propose repeating these tests recording acceleration values, in addition, to perform a detailed kinematic study of the different body regions.

By contrast, women, when they are warned of braking, register lower values of sEMG signal in the post-braking phase. This may be related to the greater relative movement between the head and torso that women experience. This lower electromyographic signal after the braking phase can indicate that women (after the initial muscle tensioning due to the warning) relax the muscles earlier and are less opposed to the inertial movement of their head with respect to the torso. This translated into injury risk, which could on the one hand imply a lower risk of muscle injury (by registering lower values of sEMG signal) but a greater risk of bone injury (by experiencing a greater relative movement of the head with respect to the torso). In any case, we can conclude that cervical behaviours, when passengers are warned, are completely opposite in women and men. These conclusions can be due to anthropometric differences. For example, women are smaller, have smaller vertebra, lower head mass, and less musculature. Consequently, women suffer a different combined response of head, neck, and torso to the emergency braking forces. Perhaps the strength of the musculature is an explanation for these differences. 

Gender and awareness-related differences in cervical muscle response have also been reported by other studies [6,9,10], while other authors considered the opposite [7,55]. Given these conflicting findings, it remains unclear if awareness of perturbation timing should be controlled.

Regarding the analysis based on the age of the volunteers, where results were divided into two age groups: less than or equal to 35 years and older than 35 years. When passengers have not been warned of the emergency braking (BT1 test), increases in the intensity of the sEMG signal of TRP muscle are observed in subjects older than 35 years of age, regardless of gender, in the post-braking phase. However, these increments are more significant in women. In the case of the braking stage, women over 35 years of age continue to experience higher sEMG signal values. However, men do not seem to experience large changes in the electromyographic signal depending on their age in the emergency braking phase. These results seem to indicate that as age increases, there is a greater risk of cervical injury, especially in the post-braking phase, when passengers are not warned about emergency braking. This may be due to the fact that as age increases, muscle mass is lost, and with it, muscle strength. This biological fact is more pronounced in women, hence the more significant increases in results.

By including age in the analysis, it continues to be maintained that greater sEMG values are registered after stopping the vehicle (post-braking stage), independently of gender and age group. On the other hand, women suffered more activity in SCM muscle than men in both tests and for all age groups. This may be due to the fact that women suffer a greater number of rebounds or these alternative neck movements are more abrupt after braking the vehicle until they manage to stabilise the neck. Therefore, they need to activate the SCM muscle more, as this is the main muscle to retain the movement of the head backwards. For their part, men under or equal to 35 years experienced more TRP sEMG signal in both phases. This could also be explained by the previous reason; since men suffer fewer rebounds in the neck, therefore, they experience less relative movement between the head and the torso during both braking phases. Thus, men seem to be mostly affected only by the first forward movement of the head, which would imply greater use of the TRP muscle.

Analysing the results when passengers are previously warned about emergency braking (BT2 test), we can also observe differences. Subjects younger than or equal to 35 experienced significant increases in the intensity of the sEMG signals of both cervical muscles, regardless of gender, in braking and post-braking phases. This could be because young people have greater muscle strength. Therefore, by being warned of braking, the youngest volunteers are able to activate the muscles more to try to stop their movement; hence, they register higher values of sEMG signal.

When women are warned (BT2 test), they experience lower values of the sEMG signal in the post-braking phase with respect to the braking stage. While, on the contrary, men show higher values in the post-braking phase, also showing more notable differences between both stages. In the case of the braking phase, women older than 35 continue to experience higher sEMG signal values. However, men do not seem to experience large changes in the electromyographic signal depending on their age in the emergency braking phase. 

In conclusion, it is remarkable that the differences in cervical muscle behaviour due to gender and age are more appreciable and significant when subjects are warned about emergency braking. In any case, the main conclusions drawn from these differences in injury patterns should be taken into account when safety systems are designed to protect all type of users.

As the main limitation of the study, it must be considered that all tests were performed within the admitted physiological limits, so that the volunteer was not subjected to levels of deceleration that could cause injuries. Authors were always focused on the volunteer’s health and keeping them safe. Therefore, in order to extrapolate the conclusions drawn here to higher levels of deceleration that could cause injuries, these tests would have to be repeated with other types of subjects, such as postmortem human surrogates, dummies, or biomechanical models; because under no circumstance can research that could damage or injury use human beings as volunteers.

## 5. Conclusions

The main contribution of this study is the performance of real environment tests carried out inside an autonomous vehicle with an AEB installed and with the collaboration of volunteers, thus increasing muscle biofidelity. This research contributes to increasing the existing state of knowledge about neck behaviour under emergency braking.

From this study, results show different cervical muscle behaviour according to gender, age, and braking phase (warning and not warning):

According to the no-warning braking phase:

- Men show greater activity in the trapezius (especially, for men under or equal to 35 years); while women activate the SCM more.
- The phase after braking seems to imply a greater risk of cervical injury, regardless of the gender.
- Subjects older than 35 years register higher TRP activity in the post-braking phase. These increments are more significant in women. Therefore, as age increases, there is a greater risk of cervical injury when there is no warning.

In case of the warning braking phase, the following could be concluded:

- These differences are more appreciable when passengers are warned about emergency braking.
- Women experienced greater cervical muscles activity in the braking phase, while men did it in the post-braking stage.
- Subjects younger than or equal to 35 experienced significant increases in the intensity of the sEMG signals of both cervical muscles, regardless of gender and the phase.
- The youngest volunteers are able to activate the muscles more to try to stabilise their movement.

Moreover, it is important to highlight those differences due to gender are more significant in the post-braking stage.

All conclusions drawn from these differences in injury patterns should be considered when safety systems are designed to protect all type of users.

## Figures and Tables

**Table 1:** Technical information of the sEMG device and the electromyography pads.

**Arduino Mega**		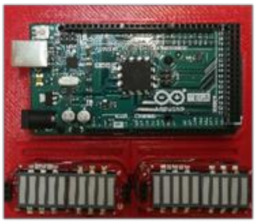
Microcontroller	ATmega2560	
Vin (V)	7–12	
Vout (V)	6–20	
Sampling rate (Hz)	1000	
sEMG low-cost sensor		
Shape/size (excl. grip)	Round/⦰ 24 mm	
Gel/adhesive/sensor area	201/251/80 mm^2^	
Sensor	Polymer Ag/AgCl	
Bandwidth (Hz)	10–400	
**Electromyography pads**		
Shape/size (excl. grip)		Round/⦰ 24 mm
Gel area		201 mm^2^
Adhesive area		251 mm^2^
Sensor area		80 mm^2^
Product thickness (adapter excluded)		1 mm
Gel characteristics		Conductive and adhesive hydrogel
Sensor		Polymer Ag/AgCl coated

**Table 2:** Technical information of the HSC.

Camera model	SONY DSC-RX0	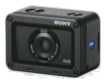
Sensor type	Sensor CMOS Exmor RS type 1.0 (13.2 mm × 8.8 mm), 3:2	
Megapixels	21.0	
Dimensions	59 mm × 29.8 mm × 40.5 mm	
Lens type	Lens ZEISS Tessar T*	
Ultra-slow motion	Up to 960/1000 fps	

**Table 3:** Technical information of the accelerometer MAHA VZM 300.

Accelerometer model	MAHA VZM 300	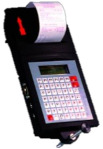
Measuring range	0–20 m/s^2^	
Internal power supply	6 V/1.8 Ah	
Measurement accuracy	±1%	
Data rate (Hz)	100	
Dimensions	245 mm × 124 mm × 55 mm	

**Table 4:** sEMG parameters for BT1 (mean of the amplitude and maximum value reached) compute in the braking and post-braking phases (phase 2 and phase 3) sorted by genre (♀ or ♂). P.P.O.M: Percentage of People Over the Mean; µ: mean value of the amplitude after normalisation (%).

			Braking		Post-Braking	
			BT1		BT1	
			µ	P.P.O.M	µ	P.P.O.M
♂	Amplitude	**TRP**	54.9	11.1	61.5	22.2
		**SCM**	21.5	11.1	24.8	16.7
♀	Amplitude	**TRP**	51.5	16.7	61.2	11.1
		**SCM**	35.1	16.7	41.6	16.7
♂	Max	**TRP**	24.3	5.6	42.6	22.2
		**SCM**	14.5	11.1	11.9	5.6
♀	Max	**TRP**	15	16.7	33.5	5.6
		**SCM**	13.5	11.1	20.6	16.7

**Table 5:** sEMG parameters for BT2 (mean of the amplitude and maximum value reached) compute in the braking and post-braking phases (phase 2 and phase 3) sorted by genre (♀ or ♂). P.P.O.M: Percentage of People Over the Mean; µ: mean value of the amplitude after normalisation (%).

			Braking		Post-Braking	
			BT2		BT2	
			µ	P.P.O.M	µ	P.P.O.M
♂	Amplitude	**TRP**	62.6	5.6	130	22.2
		**SCM**	26.4	16.7	100.4	22.2
♀	Amplitude	**TRP**	78.5	16.7	48	11.1
		**SCM**	49.7	11.1	37.2	11.1
♂	Max	**TRP**	26.1	5.6	68.9	22.2
		**SCM**	11.3	16.7	41.9	22.2
♀	Max	**TRP**	32.3	16.7	23.3	16.7
		**SCM**	14.9	16.7	9.3	5.6

**Table 6:** sEMG parameters for BT1 (mean of the amplitude and maximum value reach) compute in the braking and post-braking phases (phase 2 and phase 3) sorted by age (≤35 and >35) and genre (♀ or ♂). P.P.O.M: Percentage of People Over the Mean; µ: mean value of the amplitude after normalisation (%).

			♀			
			BT1 (Braking)		BT1 (Post-Braking)	
		Age	≤35	>35	≤35	>35
			µ	µ	µ	µ
%	Amplitude	**TRP**	28.6	82	31.3	101.2
		**SCM**	39.6	29.2	35.3	50
	Max	**TRP**	9.9	21.8	17	55.5
		**SCM**	18.8	6.5	18.4	23.4
			**♂**			
			**BT1 (Braking)**		**BT1 (Post-Braking)**	
		**Age**	**≤35**	**>35**	**≤35**	**>35**
%	Amplitude	**TRP**	54.9	54.6	59.5	75.9
		**SCM**	22.7	13.1	26.1	15.1
	Max	**TRP**	25	19.4	41	53.4
		**SCM**	15.4	8.6	12.6	7.6

**Table 7:** sEMG parameters for BT2 (mean of the amplitude and maximum value reach) compute in the braking and post-braking phases (phase 2 and phase 3) sorted by age (≤35 and >35) and genre (♀ or ♂). P.P.O.M: Percentage of People Over the Mean; µ: mean value of the amplitude after normalisation (%).

			♀			
			BT2 (Braking)		BT2 (Post-Braking)	
		Age	≤35	>35	≤35	>35
			♂			
			BT2 (Braking)		BT2 (Post-Braking)	
		Age	≤35	>35	≤35	>35
%	Amplitude	**TRP**	95.7	49.8	48.1	47.7
		**SCM**	68.9	17.6	39.5	33.4
	Max	**TRP**	34.1	29.4	28.7	14.3
		**SCM**	20.3	5.7	11.6	5.4
%	Amplitude	**TRP**	70.4	31.6	144.8	70.7
		**SCM**	32.1	3.9	123.4	8.3
	Max	**TRP**	29.6	12.4	75.2	43.9
		**SCM**	13.6	2.2	51.5	3.3
